# From Occupational Hazard to Public Health Concern: A Bibliometric and Knowledge‐Mapping Analysis of Sleep Disorders Among Nurses Worldwide (1985–2025)

**DOI:** 10.1155/jonm/7621317

**Published:** 2026-08-31

**Authors:** Sufang Lu, Jingcheng Wen, Dandan Wang, Mengjie Xu, Yongyi Dai, Lu Chen, Hongli Li, Dong Pang, Jie Li, Wen Li

**Affiliations:** ^1^ Department of Infectious Diseases, Nanjing Drum Tower Hospital, Affiliated Hospital of Medical School, Nanjing University, Jiangsu, 210008, China, nju.edu.cn; ^2^ Nursing Department, Beijing Stomatological Hospital, Capital Medical University, Beijing 100050, China, ccmu.edu.cn; ^3^ Nursing Department, Nanjing Drum Tower Hospital, Affiliated Hospital of Medical School, Nanjing University, Nanjing Jiangsu, 210008, China, nju.edu.cn; ^4^ School of Nursing, Peking University, Beijing 100083, China, pku.edu.cn

**Keywords:** bibliometric analysis, circadian disruption, CiteSpace, nurses, patient safety, shift work, sleep disorders

## Abstract

**Purpose:**

Sleep disorders among nurses are highly prevalent, affecting well‐being, clinical performance, and patient safety. A comprehensive mapping of global research trends is lacking. This study evaluates the evolution and focal points of research on sleep disorders among nurses over the last 4 decades, utilizing bibliometric analyses.

**Methods:**

We performed a bibliometric analysis of 1553 English‐language publications retrieved from the Web of Science Core Collection (WoSCC) (1985–2025). Publication trends, country and institutional contributions, collaboration networks, cocitation, and keyword co‐occurrence were analyzed.

**Results:**

Research output increased steadily, with a sharp surge after 2020, reflecting the impact of COVID‐19. The United States and China were leading contributors, but international collaboration was sparse. Core themes included insomnia and sleep quality, shift work and circadian misalignment, and psychological sequelae such as stress, depression, and burnout. Recent hotspots emphasized patient safety, measurement validation, and intervention trials.

**Conclusion:**

The field is expanding rapidly yet remains fragmented. Harmonized measurement, system‐level interventions, and stronger international collaboration are required to translate research into sustainable improvements in nurse health and patient safety.

## 1. Introduction

Sleep is a fundamental biological process that underpins cognitive performance, emotional regulation, and multisystem physiological health [1, 2]. Clinically, sleep disorders are typically manifested by impaired sleep quantity or quality, including insufficient total sleep time, reduced deep sleep, frequent nighttime awakenings, prolonged sleep latency, and early waking [3]. According to the International Classification of Sleep Disorders by the American Academy of Sleep Medicine (AASM) [4], sleep disorders are systematically classified into 6 major clinical categories: insomnia disorders, sleep‐related breathing disorders (SBDs), central excessive sleepiness disorders, circadian rhythm sleep–wake disorders, parasomnia disorders, and sleep‐related movement disorders. These problems are often associated with sleep deprivation or disrupted circadian rhythms [5].

In healthcare settings, nurses constitute the largest professional workforce and provide continuous, safety‐critical care [6]. Yet, the demands of nursing—irregular shift schedules, night work, extended hours, and sustained psychosocial stress [7, 8]—disrupt circadian alignment and sleep homeostasis, predisposing nurses to insomnia, shift work sleep disorder (SWSD) [9], excessive daytime sleepiness, and obstructive sleep apnea [10]. In a research study conducted in China, the incidence of sleep disorders among nurses was as high as 49.9% [11]. In a random survey conducted in Taiwan, the insomnia rate among nurses was 59.0% [12]. A survey conducted in Turkey on ICU nurses revealed that the incidence of sleep disorders among them was 70.5% [13]. During the coronavirus disease 2019 (COVID‐19) pandemic, a study conducted in Italy involving 1005 nurses showed that the prevalence of sleep disorders in the population was 71.4% [14]. In a meta‐analysis, the overall prevalence of sleep disorders among medical personnel was found to be 39.2% [15], which was significantly higher than that of the general population. Moreover, when compared with medical personnel in other job positions, nurses were the most likely to experience sleep disorders [16–18].

Sleep disorders compromise physiological integrity (elevated cardiovascular and metabolic risk and immune dysregulation), impair neurocognitive functions (attention, working memory, and executive control), and destabilize mood regulation [19–21]. Among nurses, these deficits reduce clinical vigilance and decision accuracy at the bedside, lower work efficiency, and heighten perceived stress, which in turn increases the likelihood of adverse emotional reactions and job burnout [22, 23]. As essential contributors to global health systems, nurses play a pivotal role in addressing contemporary healthcare demands, including aging populations, the rising prevalence of chronic diseases, and expanding needs in mental health care [24, 25]. What distinguishes nursing from many other professions is its direct and continuous impact on patient safety. Impaired sleep in nurses elevates the risk of clinical inaccuracies and, in extreme cases, may contribute to patient mortality [26–29]. These downstream effects directly impact patient safety, quality metrics, and overall health‐system efficiency.

Despite rapidly growing scholarship on nurses’ sleep health, the literature is fragmented across disorder subtypes, specialties, and regions, yielding heterogeneous methods and outcomes and obscuring the field’s intellectual structure. Traditional narrative or scoping reviews synthesize themes but are limited in mapping large‐scale publication dynamics, collaborative networks, and the emergence of new concepts.

Bibliometric analysis offers a rigorous quantitative framework to address these gaps by leveraging publication metadata [30] to (i) trace knowledge development; (ii) identify influential articles, authors, institutions, and journals; (iii) delineate thematic clusters via cocitation and keyword co‐occurrence; and (iv) detect shifting research frontiers over time. Accordingly, this study conducts a comprehensive bibliometric analysis of global research on sleep disorders among nurses.

The objectives are to (i) quantify publication volume, growth trajectories, and geographic distribution; (ii) identify leading contributors and collaboration patterns across authors, institutions, and journals; (iii) map the field’s intellectual structure and thematic evolution using cocitation and keyword co‐occurrence analyses; and (iv) detect emerging trends to inform future inquiry. The findings aim to guide researchers, administrators, and policymakers in strategic planning, catalyze international collaboration, and support targeted interventions to improve nurses’ sleep health—advancing both workforce well‐being and patient care quality.

## 2. Methods

### 2.1. Data Source and Search Strategy

Research on sleep disorders among nurses can be traced back to 1967 based on the earliest related record indexed in the Web of Science database. However, considering the consistency and completeness of bibliographic records and citation information, publications from January 1985 to August 2025 were selected for bibliometric analysis. Publications were retrieved from the Web of Science Core Collection (WoSCC) on August 23, 2025, without additional restrictions on specific indexes. The search strategy was developed after reading previously identified relevant literature and formulated based on the MeSH terms, subheadings, and subordinate terms related to “sleep disorders” and “nurses” in the PubMed database, in accordance with the search rules. The retrieval strategy was performed using the Topic (TS) field in the WoSCC. The Topic field covers titles, abstracts, author keywords, and KeyWords Plus, enabling a comprehensive identification of publications related to sleep disorders among nurses. The search formula was as follows: TS = ((“sleep disorder^∗^” OR “sleep disturbance^∗^” OR insomnia OR “poor sleep quality” OR “sleep problem^∗^” OR “sleep deprivation”) AND (nurse^∗^ OR “nursing staff” OR “registered nurse^∗^” OR “clinical nurse^∗^”)).

**FIGURE 1:**
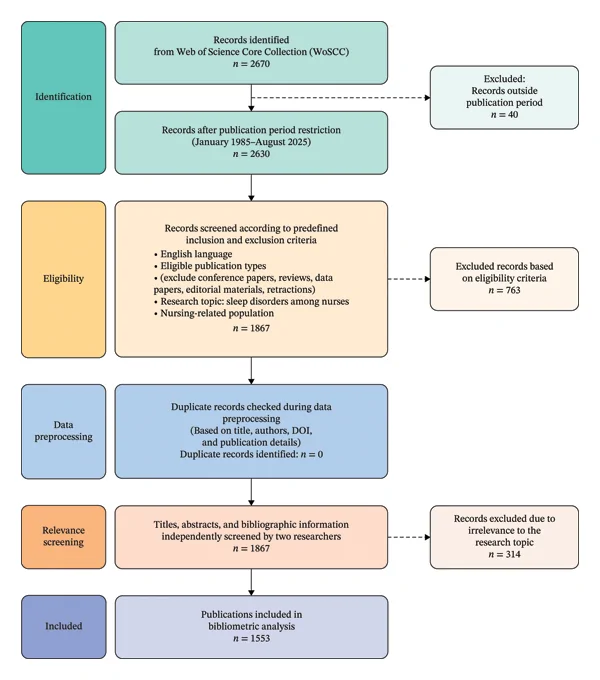
Flow diagram of literature identification, eligibility screening, and inclusion of studies on sleep disorders among nurses for bibliometric analysis.

The inclusion criteria were studies that (1) focused on sleep disorders among nurses, (2) involved nursing populations, (3) were published as papers with complete bibliographic information available in WoSCC, and (4) were written in English. Conference papers, reviews, data papers, editorial materials, and retracted publications were excluded. Early Access records were considered eligible if they met the inclusion criteria and provided sufficient bibliographic information for analysis. After the initial retrieval, all records were independently screened by two researchers according to the predefined inclusion and exclusion criteria. The titles, abstracts, and relevant bibliographic information were assessed to determine whether the records met the eligibility criteria. Any disagreements between the two researchers were resolved through discussion, and a third researcher was consulted when necessary. The detailed identification, screening, exclusion, and inclusion process is shown in Figure 1.

### 2.2. Data Analysis

CiteSpace [31], a scientific literature analysis tool, was jointly developed by Dr. Chen of Drexel University and WISE Laboratory of Dalian University of Technology. CiteSpace focuses on analyzing the knowledge embedded in the scientific literature and presenting the structure, patterns, and distribution of scientific knowledge through visual means. The term [32] CiteSpace stands for Citation Space. This type of visualization is also known as scientific knowledge mapping. To ensure clarity, we provide explanations for key terms in Table 1.

**TABLE 1:** Definitions of key terms and parameters used in CiteSpace analysis.

Parameter	Description
*Search parameters*	
TOP *N* = 50	Specifies the number of top items (e.g., keywords, authors, institutions) selected from each time slice based on their citation frequency or centrality
*K* value = 25	Used in the burst detection algorithm to identify sudden increases in the frequency of terms. Balances sensitivity and specificity in detecting bursts

*Network parameters*	
Link retaining factor (LRF) = 3.0	Determines how many links are retained when merging networks across different time slices. Higher values retain more links, preserving network continuity
Look back years (LBY) = 5.0	Specifies the number of years to look back when creating a network for a particular year, including relevant links from previous years. Five years were considered
*e* = 1	A scaling factor used in the network pruning process, affecting the stringency of the pruning algorithm

*Evaluation indices*	
Modularity value	Measures the average module value of clustering, indicating the strength of the division of the network into modules
Silhouette value	Measures the average outline value of clustering, assessing the cohesion within clusters and separation between clusters

*Centrality and frequency*	
Centrality	An important result of the keyword analysis that measures the importance of nodes in the co‐occurrence network. Nodes with high centrality are key hubs in the network
Frequency	Represents the strength of the research hotspot; higher frequency indicates a stronger hotspot

All the explanations are taken from the literature on the methodological functions of the CiteSpace knowledge graph by Chen Yue and other researchers [33, 34].

In this study, the screened publications were exported and saved in RefWorks format. Before CiteSpace analysis, data preprocessing was performed to improve the accuracy of network construction. Author, institutional, and keyword information was manually checked and standardized by merging synonymous terms, abbreviations, and different naming formats referring to the same entity or concept. For keyword analysis, terms with similar meanings were unified to avoid duplicate representation of the same research concept. The processed data were then transformed and analyzed using CiteSpace 6.2.R4 software. The software settings were as follows. The parameter time span was set as January 1985–August 2025, with each year as a time slice. The following parameters were used for network construction and visualization: TOP *N* = 50, *K* value = 25, link retaining factor (LRF) = 3.0, look back years (LBY) = 5.0, and *e* = 1. In the network pruning function settings, we ticked the “Pruning” option in the critical path (Pathfinder), “Pruning sliced networks,” and “Pruning the merged network,” and authors, institutions, and keywords were set. For the node type in the relevance analysis, we used CiteSpace’s clustering function for clustering and the log‐likelihood ratio (LLR) algorithm to extract the cluster name. After completion, the CiteSpace software provided two evaluation indices: modularity value (average module value of clustering) and silhouette value (average outline value of clustering) to evaluate whether the clustering was reasonable and valuable. After completing the clustering of this module, it was viewed in the parameter display area at the top left of the clustering map. Centrality, which is an important result of the keyword analysis, is an indicator used to measure the importance of nodes in the co‐occurrence network. Nodes with high betweenness centrality usually occupy important positions within the network and serve as bridges connecting different research themes. Frequency reflects the occurrence frequency of keywords and provides an indication of research attention toward specific topics. Keyword bursts were detected using CiteSpace burst detection. Burst strength reflects the intensity of a rapid increase in attention toward a specific keyword, while burst duration indicates the period during which the keyword exhibited a significant increase in attention.

To further clarify the methodology, additional explanations for the terms “pruning,” “node,” and “clustering function” are provided.

Pruning: In CiteSpace, pruning refers to the process of reducing the complexity of the network by removing less significant links, which helps in highlighting the most important connections. Pruning techniques such as “Pathfinder” and “Pruning sliced networks” are used to simplify the visualization and enhance the interpretability of the network structure.

Node: A node in CiteSpace represents an entity such as an author, institution, or keyword. Nodes are the fundamental units in the co‐occurrence network, and their relationships (edges) are analyzed to understand the structure and dynamics of the research field.

Clustering function: The clustering function in CiteSpace groups similar nodes into clusters based on their co‐occurrence patterns. This helps identify research themes and trends. The LLR algorithm was used to label clusters, and modularity and silhouette values were used to evaluate clustering quality.

### 2.3. Ethical Considerations

The data used in this study were obtained from the WoSCC. No patients or public contributions were involved in this research.

## 3. Results

### 3.1. Publication Output Analysis

**FIGURE 2:**
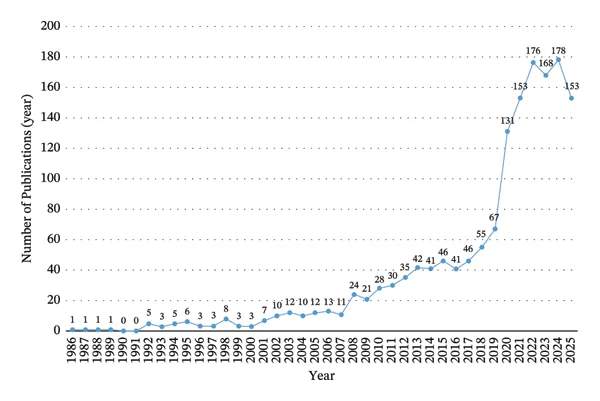
Annual publication trends of research on sleep disorders among nurses from 1985 to 2025.

A total of 2670 records were retrieved from the WoSCC, and 1553 publications were included in the final analysis after screening and preprocessing. The annual publication output provides an overview of research activity and publication trends in the field of sleep disorders among nurses. Figure 2 shows an overall increasing trend in annual publications from 1985 to August 2025. In 1985, only a very limited number of publications were identified in this field. After 2002, the annual number of publications exceeded 10 for a prolonged period. Since 2013, annual publications have exceeded 40, and since 2020, the number has exceeded 100 per year. This rapid growth indicates an accelerated expansion of research activity on sleep disorders among nurses in recent years.

### 3.2. Analysis by Country/Region and Institution

**FIGURE 3:**
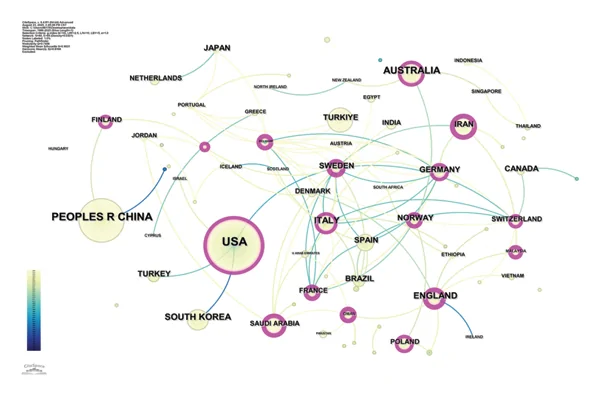
Collaboration network of countries/regions in research on sleep disorders among nurses.

**FIGURE 4:**
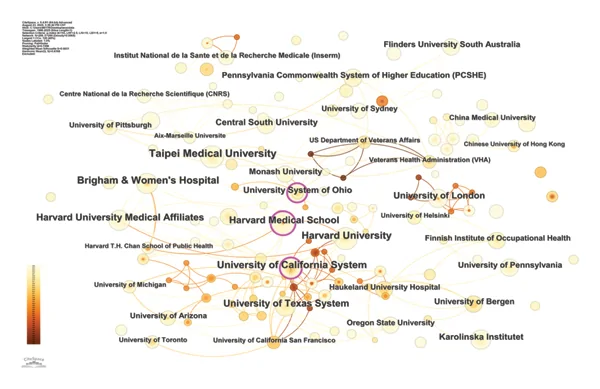
Institutional collaboration network in research on sleep disorders among nurses.

A total of 80 countries/regions and 299 institutions contributed publications on sleep disorders among nurses. The analytical profiles are shown in Figures 3 and 4. In this analysis, the node type in CiteSpace was set as “Country,” the time span was set from 1985 to 2025, the time slice was set to 1 year, and Top N was set to 50 to generate the collaboration network among countries/regions.

In the collaboration network, each node represents a country/region, and the node size reflects the number of publications contributed by that country/region. Larger nodes indicate higher publication output. A total of 80 countries/regions (*N* = 80, *E* = 95) were included in the network, with 95 collaboration links identified among these nodes. The network density was 0.0301, indicating that the overall collaboration network among countries/regions was relatively sparse.

The top 10 countries/regions according to publication output were the USA, China, Australia, England, South Korea, Türkiye/Turkey, Italy, Spain, Sweden, and Germany. The detailed publication information of the top 10 countries/regions is presented in Table 2.

**TABLE 2:** Top 10 countries/regions by publication output in research on sleep disorders among nurses.

Rank	Frequency	Centrality	Betweenness centrality	Country
1	398	0.12	1986	USA
2	340	0.08	1998	China
3	99	0.2	1997	Australia
4	78	0.54	1988	England
5	71	0	1998	South Korea
6	99	0	2005	Türkiye/Turkey
7	55	0.15	1996	Italy
8	46	0.1	2009	Spain
9	45	0.57	1994	Sweden
10	43	0.15	1998	Germany

The United States contributed the largest number of publications among all countries/regions and showed relatively extensive collaboration with other countries/regions. In addition, countries such as Italy and Germany occupied relatively central positions in the collaboration network. The United States had a higher publication output and centrality compared with other countries/regions, indicating its active participation and relatively important position in the international collaboration network of sleep disorder research among nurses. However, the overall collaboration network among countries/regions showed relatively low connectivity, suggesting that international collaboration remained limited and unevenly distributed. Further strengthening cross‐country collaboration may facilitate knowledge exchange and promote the development of research on sleep disorders among nurses.

In Figure 4, a total of 299 nodes (*N* = 299) were identified, representing institutions that published papers on sleep disorders among nurses. Overall, 290 collaboration links (*E* = 290) were generated among these institutions, and the network density was 0.0065. The relatively low network density indicates that interinstitutional collaboration was limited and that the collaboration network remained relatively dispersed. The size of each node represents the publication output of the corresponding institution, with larger nodes indicating a greater number of publications.

The top 10 institutions by publication output are presented in Table 3, including Harvard University (USA), Taipei Medical University (Taiwan), University of California System (USA), Brigham and Women’s Hospital (USA), The University of Texas System (USA), University of London (England), University System of Ohio (USA), Karolinska Institutet (Sweden), Central South University (China), and Sichuan University (China).

**TABLE 3:** Top 10 institutions by publication output in research on sleep disorders among nurses.

Rank	Frequency	Centrality	Year	Institution
1	60	0.14	1992	Harvard University
2	24	0.03	2015	Taipei Medical University
3	21	0.18	1993	University of California System
4	21	0.03	2003	Brigham and Women’s Hospital
5	17	0.08	2000	The University of Texas System
6	15	0.03	1992	University of London
7	14	0.11	2009	University System of Ohio
8	14	0	2003	Karolinska Institutet
9	13	0	2020	Central South University
10	12	0.05	2021	Sichuan University

Figure 4 shows several collaborative clusters among institutions. Some institutions with higher publication output, such as Harvard University and the University of Toronto, were located in relatively central positions within the collaboration network, indicating their active participation and stronger connectivity within this research field. However, the overall institutional collaboration network remained relatively dispersed, suggesting that collaboration among institutions was not evenly distributed. Increasing cooperation among institutions from different regions may contribute to broader research exchanges and the further development of studies on sleep disorders among nurses.

### 3.3. Analysis of Cocited Publications

**FIGURE 5:**
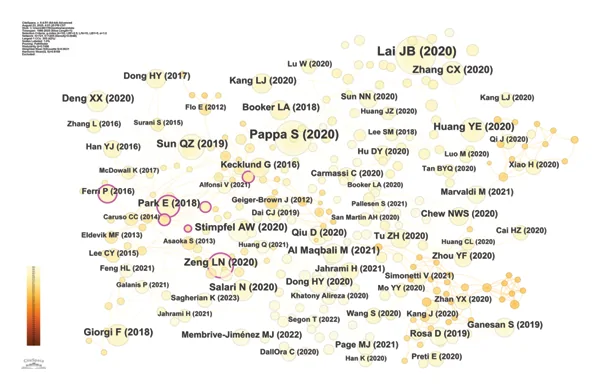
Cocitation network of cited publications in research on sleep disorders among nurses.

Cocitation analysis provided a deeper grasp of the research on this topic. The frequency with which the academic literature is cited can be used as a basis for determining its value. Highly cited literature serves as the foundation for the development of a discipline, making it an essential component in the advancement of a given field. A total of 721 nodes (*N* = 721) are present in Figure 5, representing 721 cited studies, and a total of 1205 connecting lines (*E* = 1205) were generated between the cited studies, representing the connection between each cited publication. The density of the network was 0.0046.

Based on cocitation frequency, several highly cocited publications were identified as important references in research on sleep disorders among nurses. The most frequently cocited publication was a research article [35], with a citation frequency of 86 (Figure 5; Table 4). This study investigated factors associated with mental health outcomes among healthcare workers infected with COVID‐19. As of the latest retrieval date, this publication had received 4470 citations. The second most frequently cocited publication was the systematic review and meta‐analysis by Pappa et al. [36], which focused on the prevalence of depression, anxiety, and insomnia among healthcare workers during the COVID‐19 pandemic, with a total of 72 cocitations. The study by Sun QZ published in the Journal of Nursing Management in 2018 named “Sleep problems in shift nurses: A brief review and recommendations at both individual and institutional level” had a total cocitation frequency of 35. The study was a narrative review showing us that the sleep patterns, sleep quality of shift nurses, the implemented intervention measures in the nursing settings, and solutions have been proposed for individual nurses, nurse managers, and healthcare institutions to improve the sleep health of shift nurses. Huang YE et al.’s survey [37] titled “Generalized anxiety disorder, depressive symptoms and sleep quality during COVID‐19 outbreak in China: a web‐based cross‐sectional survey” also received a high cocitation frequency. As of the date of the most recent retrieval, the study has been cited 2355 times. This study shows that compared with other occupational groups, medical staff are more likely to have poor sleep quality and mental health problems (*p* < 0.05). The fifth most cocited study [38] was “Survey of insomnia and related social psychological factors among medical staff involved in the 2019 novel coronavirus disease outbreak,” which demonstrated the prevalence of insomnia among medical staff in hospitals during the COVID‐19 pandemic and identified the associated social and psychological factors. The survey found that among 1563 healthcare workers, 564 individuals (accounting for 36.1%) reported having insomnia symptoms based on the Insomnia Severity Index (ISI) (with a total score of 8 or higher). The top 10 highly cocited publications are presented in Table 4.

**TABLE 4:** Top 10 highly cocited publications in research on sleep disorders among nurses.

Frequency	Burst	Centrality	Name	Source	DOI
86	9.23	0.00	Lai JB (2020)	JAMA Network Open	10.1001/jamanetworkopen.2020.3976
72	7.89	0.05	Pappa S (2020)	Brain, Behavior, and Immunity	10.1016/j.bbi.2020.05.026
35	3.84	0.01	Sun QZ (2019)	Journal of Nursing Management	10.1111/jonm.12656
33	9.74	0.03	Huang YE (2020)	Psychiatry Research	10.1016/j.psychres.2020.112954
32	6.40	0.02	Zhang CX (2020)	Frontiers in Psychiatry	10.3389/fpsyt.2020.00306
30	0.00	0.10	Zeng LN (2020)	Behavioral Sleep Medicine	10.1080/15402002.2019.1677233
30	12.76	0.10‐	Stimpfel AW (2020)	Sleep Health	10.1016/j.sleh.2019.11.001
28	6.85	0.02	Giorgi F (2018)	Journal of Advanced Nursing	10.1111/jan.13484
26	4.70	0.05	Al Maqbali M (2021)	Journal of Psychosomatic Research	10.1016/j.jpsychores.2020.110343
26	8.86	0.04	Salari N (2020)	Globalization and Health	10.1186/s12992‐020‐00620‐0

### 3.4. Keyword Analysis

#### 3.4.1. Keyword Co‐Occurrence Analysis

**FIGURE 6:**
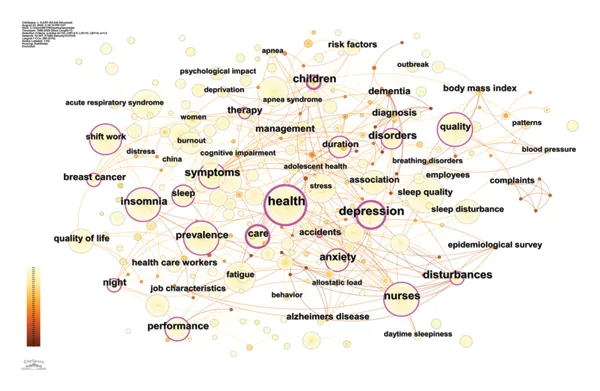
Keyword co‐occurrence network in research on sleep disorders among nurses.

Keyword mapping provides insights into frequently discussed topics and thematic structures within a research field. We analyzed the keywords of this topic using CiteSpace software, setting the node type as “Keyword”, the time span as 1985–2025, the time slice as every 1 year, and Top N as 50. The keyword co‐occurrence map is drawn Figure 6. As shown in Figure 6, each node represents a keyword. A total of 307 nodes representing 307 keywords appeared in the map. The larger the area of a node, the higher the frequency of the keyword in the literature, reflecting frequently discussed topics within the field.

The top 10 most frequent keywords were “health,” “insomnia,” “sleep quality,” “prevalence,” “quality,” “nurses,” “shift work,” “stress,” “impact,” and “depression,” with 267, 196, 195, 194, 182, 180, 175, 152, 149, and 147 occurrences, respectively.

Centrality is also known as betweenness centrality. Higher centrality indicates that a keyword occupies a more central position within the keyword co‐occurrence network and has stronger connections with other keywords. When the mediated centrality of the keyword was > 0.1 [39], it was considered as a key node. According to the data in Table 5, the keywords with centrality greater than 0.1 were “health,” “insomnia,” “prevalence”, “quality”, “nurses”, “shift work,” and “depression,” suggesting that these keywords played relatively important structural roles in the keyword co‐occurrence network.

**TABLE 5:** Top 10 keywords by frequency and betweenness centrality.

Rank	Frequency	Centrality	Year	Keyword
1	267	**0.31**	1994	Health
2	196	**0.13**	1999	Insomnia
3	195	0.07	2002	Sleep quality
4	194	**0.15**	1992	Prevalence
5	182	**0.15**	2007	Quality
6	180	**0.19**	1999	Nurses
7	175	**0.12**	1995	Shift work
8	152	0.02	2002	Stress
9	149	0.04	1995	Impact
10	147	**0.2**	1992	Depression

*Note:* Bold font: The centrality is greater than 0.1.

#### 3.4.2. Keyword Cluster Analysis

**FIGURE 7:**
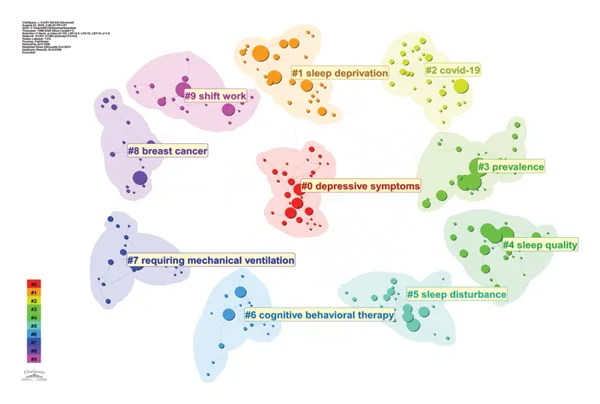
Keyword clustering map based on LLR analysis.

On the basis of the keyword clustering map, the LLR clustering algorithm was chosen to start the keyword cluster analysis. A total of 10 large and small clusters were generated, resulting in the final diagram of the keyword clustering analysis (Figure 7).

Modularity refers to the cluster modularity value, called the “*Q*” value. *Q* > 0.3 means that the structure of the cluster is significant [40]. The *Q* value of this cluster was 0.7456; therefore, the structure of the cluster was considered significant. The *S* value is the average profile value of the clusters, indicating their homogeneity. Usually, when *S* > 0.5, the clustering is considered reasonable [41]. The average profile value of all clusters was 0.8168. Cluster analysis can more clearly show the research topics in related fields than keyword mapping. Figure 7 shows that the topics can be divided into 10 clusters, as described in Table 6.

**TABLE 6:** Information of keyword clusters identified by CiteSpace analysis.

ClusterID	Size	Silhouette	Mean (year)	Label (LSI)	Label (LLR)
0	31	0.864	2006	sleep disturbance; work environment; health‐promoting behaviors; quality; disturbance | sleep quality; relaxation exercises; occupational burnout; risk assessment; disturbances	depressive symptoms (23.57, 1.0E − 4); adolescents (14.74, 0.001); patterns (10.15, 0.005); disorders (9.66, 0.005); parent (9.64, 0.005)
1	29	0.903	2010	sleep deprivation; psychiatric nurses; occupational burnout; disturbances; managing fatigue | sleep disorders; emergency healthcare workers; mental fatigue; physical fatigue; shift work	sleep deprivation (55.11, 1.0E − 4); performance (21.94, 1.0E − 4); sleep quality (12.99, 0.001); shift work schedule (12.12, 0.001); mental health (9.22, 0.005)
2	28	0.926	2018	healthcare workers; sleep quality; systematic review; anxiety; work condition | mental health; health profession; sleep disturbance; stress disorders; hospital staff	covid‐19 (51.73, 1.0E − 4); healthcare workers (32.35, 1.0E − 4); covid‐19 pandemic (27.06, 1.0E − 4); mental health (15.75, 1.0E − 4); pandemic (13.77, 0.001)
3	27	0.889	2009	sleep quality; non‐rapid eye movement; sleep disturbances; depressive symptoms; healthcare worker | sleep disorders; excessive daytime sleepiness; multiple sclerosis; eortc qlq‐c30; parental cancer	prevalence (9.8, 0.005); workplace (9.58, 0.005); health (8.72, 0.005); smartphone (8.34, 0.005); sleep health (8.34, 0.005)
4	26	0.889	2009	sleep quality; non‐rapid eye movement; outpatient emergencies; atopic dermatitis; work environment | sleep disturbance; work environment; health‐promoting behaviors; major depressive disorder; critical care nursing	sleep quality (98.74, 1.0E − 4); randomized controlled trial (8.57, 0.005); shift workers (8.45, 0.005); job demand (8.45, 0.005); systemic lupus erythematosus (8.45, 0.005)
5	25	0.851	2012	sleep disturbance; shift‐work tolerance; personal traits; coping strategies; work‐life balance | sleep quality; body mass index; body composition; waist circumference; Pittsburgh sleep quality index	sleep disturbance (32.23, 1.0E − 4); older adults (11.67, 0.001); primary health care (9.45, 0.005); adult nursing (9.45, 0.005); type 2 diabetes (9.45, 0.005)
6	19	0.936	2002	sleep disturbance; advanced practice; integrated care; nurses practitioners; women health | sleep quality; critical care nursing; coronary artery bypass; nurse‐led intervention; sleep disorders	covid‐19 (8.86, 0.005); cognitive behavioral therapy (8.67, 0.005); primary care (7.26, 0.01); pain (6.42, 0.05); older people (6.19, 0.05)
7	18	0.882	2007	sleep disturbance; psychological distress; junior doctors; symptom trajectory; women health initiative | sleep problems; healthcare professionals; emotional burden; coping strategies; quality	requiring mechanical ventilation (11.18, 0.001); critical illness (11.18, 0.001); disruption (11.18, 0.001); workaholism (11.18, 0.001); delirium (10.65, 0.005)
8	17	0.937	2002	breast cancer; mental health; telehealth programs; critical care; cognitive intervention | sleep quality; female workers; self‐perceived health; sleep disturbance; depressed mood	breast cancer (53.83, 1.0E − 4); quality of life (37.21, 1.0E − 4); cancer (14.46, 0.001); nurses (14.05, 0.001); radiation therapy (11.25, 0.001)
9	17	0.96	2013	sleep disorders; sleep quality; health professionals; pediatric intensive care unit; sleep impairment | shift work disorder; circadian rhythm; sleep reactivity; atypical work hours; circadian misalignment	shift work (59.61, 1.0E − 4); sleep disorders (39.15, 1.0E − 4); night work (17.52, 1.0E − 4); sleep disturbance (11.56, 0.001); mental health (11.32, 0.001)

#### 3.4.3. Keyword Burst Analysis

**FIGURE 8:**
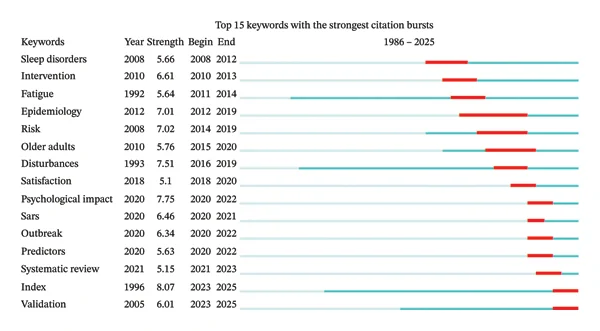
Keyword burst detection map showing emerging research topics.

Keyword burst analysis was conducted to identify emerging research topics and changes in research attention over time. From 2008 to 2012, the keyword “sleep disorders” showed a citation burst lasting 4 years, with a burst strength of 5.66. The keyword “fatigue” showed a burst from 2011 to 2014, with a strength of 5.64. From 2020 to 2022, the keywords “psychological impact” and “outbreak” showed citation bursts, with strengths of 7.75 and 6.34, respectively. The keywords “index” and “validation” showed the strongest recent bursts from 2023 to 2025, with burst strengths of 8.07 and 6.01, respectively. These emerging keywords reflect recent changes in research attention and may provide insights into evolving themes in the study of sleep disorders among nurses (Figure 8).

### 3.5. Analysis of the Timeline and Time‐Zone Map

**FIGURE 9:**
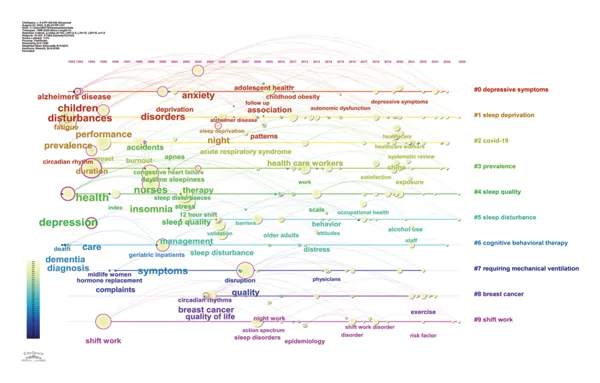
Timeline view of keyword evolution in research on sleep disorders among nurses.

**FIGURE 10:**
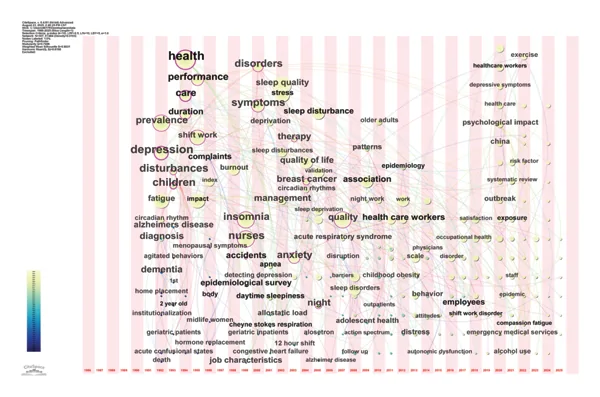
Time‐zone view of keyword evolution in research on sleep disorders among nurses.

Using CiteSpace, keyword timeline and time‐zone views were generated based on the keyword co‐occurrence network to explore the temporal evolution of research topics (Figures 9 and 10). The timeline and time‐zone maps illustrate changes in keyword occurrence and connections among keywords from 1985 to 2025.

According to Figures 9 and 10, the evolution of research themes related to sleep disorders among nurses can be observed over different periods. In 1985, keywords such as “health,” “depression,” and “disturbance” appeared frequently, reflecting early research attention to psychological and sleep‐related problems. The emergence of “insomnia” in 1999 showed relatively high betweenness centrality, indicating its important position within the keyword network. Between 2000 and 2005, keywords including “anxiety” and “quality of life” showed strong connections with other research topics, suggesting an expansion of research focus during this period. During the early development stage (1992–2012), frequently occurring keywords mainly focused on “symptoms,” “sleep quality,” “anxiety,” “therapy,” “sleep disturbance,” and “stress,” indicating that early studies primarily explored sleep‐related symptoms, psychological factors, and potential interventions among nurses. Over time, additional keywords emerged, reflecting the gradual diversification of research topics. From 2012 to 2019, keywords such as “scale,” “occupational health,” and “shift work disorder” appeared, indicating increased attention to measurement tools, occupational factors, and shift‐related sleep problems. More recent keywords, including “alcohol use,” “psychological impact,” “exposure,” and “risk factor,” further expanded the scope of research topics. Since 2020, keywords such as “outbreak,” “exposure,” “epidemiology,” and “emergency medical services” have appeared frequently. These emerging terms suggest that COVID‐19–related occupational stressors and healthcare emergency contexts became important research topics during this period.

The keyword timeline (Figure 9) and keyword time zone (Figure 10) were combined for comparison and analysis, which showed the evolution of keyword research themes on this topic and the basic path of evolution along the direction of keywords.

## 4. Discussion

High‐quality sleep is a necessary condition for a good life and work. The sleep quality of nurses is closely related to the quality of nursing and the prognosis of patients’ diseases [42]. This study conducted a bibliometric analysis of global research on sleep disorders among nurses from 1985 to 2025, including 1553 publications, and visualized the development trends, knowledge structure, and thematic evolution of this field. The findings showed a continuous increase in publication output over the study period. Annual publications exceeded 100 after 2020, indicating a rapid expansion of research activity in this area in recent years. This increase coincided with the COVID‐19 pandemic period, during which research attention to healthcare workers’ sleep problems and related psychological factors increased. The emerging keywords identified in the keyword burst analysis, including “outbreak,” “psychological impact,” and “exposure,” further reflected the increased attention to pandemic‐related occupational challenges. In addition, the inclusion of publications from 80 countries/regions and 299 institutions provided a broad basis for analyzing international and institutional collaboration patterns.

### 4.1. Current Status of Global Research on Sleep Disorder Among Nurses

Our bibliometric mapping (1985–2025; *n* = 1553) shows a sustained growth trajectory with a marked increase in publication output around 2020. Annual output was generally < 40 papers before 2013, surpassed 40 thereafter, and exceeded 100 per year after 2020. This increase indicates growing scholarly attention to nurse sleep health and its relationship with occupational health and patient safety [43].

Geographically, 80 countries/regions have contributed to the field. The United States and China were among the leading contributors; the United States showed the highest betweenness centrality in the international coauthorship network, whereas China demonstrated substantial publication output growth. These patterns may reflect differences in research capacity, healthcare systems, and academic collaboration structures among countries/regions [44, 45]. The U.S. National Institute for Occupational Safety and Health (NIOSH) has highlighted fatigue and shift work as important occupational health concerns in healthcare [46, 47], and relevant workforce policies in China have provided a broader context for increasing attention to nurses’ sleep and mental health during and after the COVID‐19 pandemic [48, 49]. Beyond individual countries, international organizations have provided a broader policy context related to healthcare workforce well‐being. The World Health Organization (WHO) has emphasized health workforce well‐being as a pillar of health‐system resilience, introducing recommendations on “Health Worker Safety: A Priority for Patient Safety” in 2020, which explicitly identified fatigue and sleep disruption as threats to both staff health and patient outcomes [50–52]. Similarly, the International Labour Organization (ILO) has developed global frameworks for regulating working hours and protecting night‐shift workers [53, 54], which provide an international policy context for addressing nurse sleep disorders. These global initiatives provide a relevant context for understanding the increasing attention to occupational health, patient safety, and workforce well‐being. However, collaboration remains relatively limited: The country‐level network contains few links (density = 0.0301), and institutional collaboration is even more dispersed (299 institutions; density = 0.0065). Such fragmentation constrains multicenter designs and limits cross‐cultural external validity.

The thematic structure is relatively stable yet continues to evolve. High‐frequency and high‐centrality keywords mainly focused on insomnia/sleep quality, shift work–related factors, and psychological outcomes. Since 2020, there has been a visible broadening toward patient‐safety endpoints (e.g., medical errors and needlestick injuries) [27, 55] and toward intervention‐oriented terms (e.g., cognitive–behavioral therapy for insomnia [CBT‐I] and randomized trials) [56, 57]. Nevertheless, measurement practice remains heterogeneous: Widely used instruments (e.g., PSQI and sleepiness scales) [58] are not shift‐pattern–sensitive, and validation specific to SWSD is limited—an issue reflected in the rising prominence of “index/validation” terms in recent years [59, 60].

In sum, the current landscape is characterized by rapid quantitative expansion, increasing international participation, and growing attention to workforce health, while also showing relatively low‐density collaboration networks, methodological heterogeneity, and variation in measurement approaches. Future research may benefit from stronger cross‐country and cross‐institutional collaboration, harmonized definitions and measurement approaches tailored to shift work, and larger multicenter studies integrating psychological, patient‐safety, and economic outcomes.

### 4.2. Research Focuses on Sleep Disorder Among Nurses

Research focuses were identified based on high‐frequency and high‐centrality keywords, which provide insights into frequently discussed topics and thematic structures within the identified publications. The analysis and visualization of keywords helped characterize major research themes and their temporal evolution. Herein, we summarize the major research themes related to sleep disorders among nurses.

#### 4.2.1. Insomnia and Subjective Sleep Quality

High‐frequency, high‐centrality terms (insomnia, sleep quality, and prevalence) indicate that epidemiological characterization of sleep disturbance, predominantly using subjective instruments, remains one of the major research areas in this field. Across nurse cohorts, point‐prevalence estimates typically fall in the 40%–70% range [61–63], but they vary systematically [12, 58] by instrument (e.g., PSQI and ISI), cutoff (e.g., PSQI ≥ 5 vs. ≥ 7), recall window, clinical setting (ICU/ED vs. general wards), and shift pattern exposure [64]. Methodologically, most studies are cross‐sectional and rely on single‐time, self‐reported measures, which increases susceptibility to common‐method bias and limits inference about temporality. Nevertheless, convergent associations are consistently observed: Poorer subjective sleep quality and higher insomnia severity correlate with diminished vigilance and psychomotor performance, greater negative affect and perceived stress, and downstream occupational sequelae (e.g., near‐misses and reduced task efficiency) [65–68]. This trend is consistent with increasing recognition of nurse sleep health as an issue related to both individual well‐being and patient safety.

#### 4.2.2. Shift Work and Circadian Misalignment

Keyword co‐occurrence and clustering analyses identified shift work and circadian rhythm as prominent themes within this research field: In our map, shift work ranks among the top‐frequency terms (175 occurrences) and meets the key‐node criterion for centrality (≥ 0.10), suggesting its central position within the keyword co‐occurrence network. Combined cluster labels explicitly bind “shift work disorder” and “circadian misalignment,” reflecting a consolidated thematic core rather than isolated subtopics.

Mechanistically, shift schedules outside the diurnal window disrupt endogenous circadian timing that regulates sleep–wake propensity, hormone release, and cardiovascular activity, thereby impairing physiological homeostasis and increasing susceptibility to sleep pathology [5, 69]. Empirical syntheses within the nursing context further specify exposure–response features at both individual and organizational levels: Rapid rotations, short inter‐shift intervals, irregular patterns, and consecutive night duties accumulate sleep debt and destabilize melatonin‐mediated rhythms [70, 71].

Operational consequences have been quantified with time‐of‐day and shift‐length sensitivities. Performance declines between 04:00 and 08:00, a window coinciding with elevated medical‐error incidence during night work; shifts > 12 h reduce vigilance and are associated with approximately two‐fold higher odds of errors relative to shorter shifts, with concomitant decrements in job satisfaction [72–74]. These findings align with broader evidence linking shift work to cardiometabolic and neurocognitive risk but are distinctive in their specificity to clinical safety endpoints and their anchoring in circadian timing [69, 75]. Together, the bibliometric centrality of shift work/circadian rhythm, the physiologic rationale for rhythm disruption, and the quantified safety penalties at circadian low points provide a comprehensive perspective on how scheduling practices may influence nurse sleep health and patient safety.

#### 4.2.3. Psychological Comorbidities and Occupational Burnout

Across datasets, mental health–related terms (e.g., stress, depression, and burnout) frequently appeared as high‐frequency keywords in nurse sleep research, highlighting the close association between sleep disturbance and psychological outcomes reported in previous studies [76, 77]. Large cohort surveys further document the co‐occurrence of anxiety, depression, and insomnia in frontline nurses. A cohort study conducted in Taiwan (*n* = 46,120) reported that the adjusted hazard ratios (HRs) for treated anxiety, depression, and insomnia among nurses were 0.91 (95% confidence interval [CI], 0.88–0.95), 0.59 (95% CI, 0.55–0.63), and 1.43 (95% CI, 1.38–1.48), respectively, reinforcing the syndromic clustering of sleep and mood symptoms in real‐world clinical environments [78]. Prospective and cross‐sectional studies have reported complex associations among sleep disruption, perceived stress, negative affect, burnout, and occupational performance.

#### 4.2.4. COVID‐19 and Public Health Crises

Bibliometric findings indicate that public health emergencies became an important research context for nurses’ sleep studies. In our timeline and burst analyses, COVID‐19, pandemic, and healthcare workers emerged as salient burst terms with high strengths (e.g., “covid‐19” strength = 51.73; “healthcare workers” and “pandemic” also prominent), reflecting increased attention to crisis‐related sleep research after 2020. Previous studies and related literature also highlighted “outbreak/healthcare workers/mental health” as international bursts during 2021–2023, supporting the field‐wide pivot to pandemic contexts. Empirical evidence within the highly cited corpus substantiates this pivot. A JAMA Network Open multicenter study of healthcare workers during COVID‐19 and a meta‐analysis of depression, anxiety, and insomnia in healthcare workers anchor the cocitation network in our dataset, while large web‐based surveys in China highlight the co‐occurrence of poor sleep with elevated psychological distress in medical staff [35–37]. Mechanistically and clinically, studies conducted during the pandemic converge on a mediational profile whereby extreme workload and stress exposure are linked to anxiety, depression, and insomnia among frontline staff; an insomnia prevalence of 36.1% (ISI ≥ 8) was documented in 1563 hospital workers early in the outbreak, and medical staff were significantly more likely than other occupational groups to report poor sleep quality and mental health problems [79]. Collectively, these findings suggest that sleep disturbance represents an important indicator of occupational strain during public health crises and highlight the relevance of nurse sleep health to patient safety and healthcare workforce well‐being.

#### 4.2.5. Measurement and Intervention Exploration

Recent bursts of keywords such as “index,” “validation,” and “randomized controlled trial” suggest increasing attention to measurement refinement and intervention evaluation in nurse sleep research, extending beyond descriptive epidemiological studies. While early studies were largely confined to prevalence surveys, more recent work has sought to validate instruments specifically in nursing cohorts and to test the effectiveness of interventions such as CBT‐I [80], digital or group‐based sleep education [81], and bright‐light exposure during night shifts [57, 82]. However, widely used tools such as the PSQI and BSWSQ remain limited in their ability to differentiate shift‐pattern heterogeneity or to attribute symptoms directly to shift work, which partly explains the inconsistency of prevalence estimates across settings [83–86]. Moreover, many existing intervention trials are often single‐site, short‐term, and reliant on self‐reported outcomes, making their generalizability uncertain. At the organizational level, strategies that are frequently recommended in narrative reports—such as fatigue risk management systems [87], forward‐rotating schedules [88], and protected rest breaks [89]—have not yet been systematically tested in large, multicenter nurse populations. This difference between growing psychometric and individual‐level intervention research and the limited evaluation of system‐level strategies indicates an important research gap. Future studies incorporating harmonized, shift‐sensitive measurement frameworks and rigorous nurse‐specific organizational trials may help improve the translation of methodological advances into scalable strategies for workforce safety and patient care quality.

### 4.3. Future Research on Sleep Disorder Among Nurses

The bibliometric patterns identified in this study suggest several areas that warrant further investigation in future research. First, future research may benefit from further mechanistic investigations to complement existing descriptive evidence. Current evidence has associated shift work and extended working hours with circadian disruption and sleep problems, while the underlying biological mechanisms, such as neuroendocrine alterations, immune dysregulation, and cardiometabolic consequences, remain to be further clarified in nurse populations. Mechanistic studies integrating physiological monitoring with psychosocial assessment may help to better understand the relationships between occupational exposures, sleep problems, and health outcomes [90, 91]. Second, measurement frameworks require harmonization and shift‐sensitive adaptation. The heterogeneity of prevalence estimates partly reflects inconsistent use of tools and cutoffs. Although PSQI and BSWSQ are widely applied, they cannot capture shift‐pattern heterogeneity or provide causal attribution to work schedules [92, 93]. Future studies should prioritize the validation of nurse‐specific instruments, incorporate objective data from actigraphy and duty rosters, and adopt standardized reporting of shift geometry (e.g., rotation speed, consecutive nights, and intershift intervals) [94]. Such methodological improvements may facilitate cross‐study comparability and future meta‐analytic synthesis. Third, intervention research could be further expanded beyond individual‐level approaches toward organizational and system‐level strategies. Large‐scale randomized or quasiexperimental studies evaluating these strategies are needed to better assess their effectiveness, cost‐effectiveness, sustainability, and potential impact on nurse well‐being and patient safety outcomes. Fourth, greater attention to underrepresented populations and contextual diversity is needed. Many existing studies have focused on hospital‐based female nurses, leaving other groups, including male nurses, mid‐to‐late career staff, and nurses working in primary care or resource‐constrained settings, less studied. Comparative and cross‐cultural research may help distinguish common and context‐specific factors influencing nurse sleep health while addressing equity in occupational health [95]. Finally, international collaboration and data‐sharing infrastructure must be strengthened. Our analysis revealed that the global research network remains relatively dispersed, with limited collaboration density across countries and institutions. Establishing research consortia, developing data‐sharing platforms, and promoting multicenter prospective cohorts may contribute to generating more comprehensive and generalizable evidence. Such initiatives should align with the WHO’s call to prioritize health worker safety as a prerequisite for patient safety, thereby integrating sleep health into broader agendas of workforce resilience and health‐system performance [96].

In summary, advancing research on nurse sleep disorders may benefit from moving beyond predominantly cross‐sectional investigations toward more integrated, multilevel, and internationally coordinated research programs. Through mechanistic investigations, measurement harmonization, system‐level intervention studies, inclusion of diverse populations, and enhanced global collaboration, future research may generate more actionable evidence to improve workforce sustainability and healthcare quality.

## 5. Limitations

Inevitably, this study has some limitations that should be acknowledged. First, this study was based on the WoSCC, which was selected because it provides standardized bibliographic records and citation information suitable for CiteSpace‐based bibliometric analysis. However, the use of a single database may result in the omission of relevant studies indexed in other databases. In addition, this study included only English‐language publications, which may introduce language bias and potentially lead to underrepresentation of nursing research from regions where non‐English publications are more common. Therefore, the geographic distribution and collaboration patterns identified in this study should be interpreted within the scope of the selected database and language criteria. Future studies incorporating multiple databases and multilingual publications may provide a more comprehensive understanding of global research trends. Second, bibliometrics depicts structures and trends, but it cannot replace the evaluation of the quality of individual studies and the strength of causal identification; therefore, it still needs to be mutually verified with systematic reviews/mixtures and original studies to form an “evidence triangle”. In the future, we will further expand our data sources, standardize our keywords, and improve research methods to help us improve the overall quality of our data and the accuracy of our predictions.

## 6. Conclusion

In this bibliometric analysis, we examined the evolution of sleep disorders research among nurses from 1985 to 2025 and analyzed 1553 publications. The findings demonstrated steady growth in research output, with a marked increase after 2020, reflecting growing attention to nurse sleep health, occupational well‐being, and patient safety.

The major research themes identified in this field include insomnia and sleep quality, shift work and circadian misalignment, and psychological and occupational outcomes, while recent studies have increasingly explored safety‐related outcomes and intervention approaches. However, methodological heterogeneity, relatively limited international collaboration, variation in measurement approaches, and insufficient evaluation of system‐level interventions remain important areas for further investigation. Some populations, including male nurses, mid‐to‐late‐career nurses, and nurses working in primary care settings, remain less represented in existing research.

Future research may benefit from harmonized, shift‐specific measurement approaches, further investigation of underlying mechanisms, rigorous evaluation of organizational interventions, and enhanced international collaboration. These efforts may contribute to generating more comprehensive evidence to support nurse well‐being and healthcare quality improvement.

## Author Contributions

Wen Li and Jingcheng Wen: conceptualization, data collection, data curation, formal analysis, investigation, methodology, software, validation, visualization, writing–original draft, and writing–review and editing.

Dandan Wang, Yongyi Dai, and Mengjie Xu: data curation, software, validation, writing–original draft, and writing–review and editing.

Jie Li, Lu Chen, Dong Pang, and Hongli Li: project administration, supervision, and writing–review and editing.

Sufang Lu: conceptualization, project administration, supervision, and writing–review and editing.

Sufang Lu, Jingcheng Wen, and Dandan Wang are co‐first authors and have contributed equally to this article. Lu Chen, Jie Li, and Wen Li are co‐corresponding authors and are responsible for correspondence regarding this article.

## Funding

This research was funded by the Science Foundation of Nanjing Drum Tower Hospital (Approval No. 2024‐DF‐03) and the Innovative Drug Research and Development—National Science and Technology Major Project (2026ZD1805104).

## Disclosure

All authors read and approved the final manuscript.

## Ethics Statement

This study is a secondary analysis of existing literature from an open database and does not involve the ethical review process.

## Conflicts of Interest

The authors declare no conflicts of interest.

## Data Availability

The data supporting the findings of this study are available from the corresponding author upon reasonable request.
